# A detergent-free strategy for the reconstitution of active enzyme complexes from native biological membranes into nanoscale discs

**DOI:** 10.1186/1472-6750-13-41

**Published:** 2013-05-11

**Authors:** Ashley R Long, Catherine C O’Brien, Ketan Malhotra, Christine T Schwall, Arlene D Albert, Anthony Watts, Nathan N Alder

**Affiliations:** 1 Department of Molecular and Cell Biology, University of Connecticut, 91 North Eagleville Rd, Storrs, Connecticut 06269, USA; 2 Department of Biochemistry, Biomembrane Structure Unit, University of Oxford, Oxford, OX1 3QU, UK

**Keywords:** Styrene-maleic acid, Copolymer, Amphipols, Membrane proteins, Nanoscale model membranes, Mitochondria, Lipodisqs®

## Abstract

**Background:**

The reconstitution of membrane proteins and complexes into nanoscale lipid bilayer structures has contributed significantly to biochemical and biophysical analyses. Current methods for performing such reconstitutions entail an initial detergent-mediated step to solubilize and isolate membrane proteins. Exposure to detergents, however, can destabilize many membrane proteins and result in a loss of function. Amphipathic copolymers have recently been used to stabilize membrane proteins and complexes following suitable detergent extraction. However, the ability of these copolymers to extract proteins directly from native lipid bilayers for subsequent reconstitution and characterization has not been explored.

**Results:**

The styrene-maleic acid (SMA) copolymer effectively solubilized membranes of isolated mitochondria and extracted protein complexes. Membrane complexes were reconstituted into polymer-bound nanoscale discs along with endogenous lipids. Using respiratory Complex IV as a model, these particles were shown to maintain the enzymatic activity of multicomponent electron transporting complexes.

**Conclusions:**

We report a novel process for reconstituting fully operational protein complexes directly from cellular membranes into nanoscale lipid bilayers using the SMA copolymer. This facile, single-step strategy obviates the requirement for detergents and yields membrane complexes suitable for structural and functional studies.

## Background

Integral membrane proteins are vital to cellular function. They represent nearly a third of all gene products and account for roughly half of all current pharmaceutical targets [1,2]. These proteins are embedded in the membrane bilayer, which supports the lipid and protein interactions essential for protein function and structural stability. The transmembrane segments of integral membrane proteins are enriched in hydrophobic side chains that are in direct contact with lipid acyl chains or with the nonpolar surfaces of other transmembrane segments. Without suitable detergents, membrane proteins denature or aggregate during extraction procedures. Therefore, solution-based analysis of isolated membrane proteins and protein complexes requires an experimental means of shielding their hydrophobic surfaces from water.

Detergent solubilization is commonly used to isolate and characterize membrane proteins. Yet the identification of a detergent that is compatible with a given target protein can be a challenge. More critically, detergent micelles are often unsatisfactory substitutes for lamellar membranes. Native lipid bilayers present specific physical and chemical properties to an integral membrane protein in the interfacial and nonpolar regions and generate an environment that is critical for membrane protein function, stability and folding [3-6]. Such an environment is not well represented in micellar structures. In addition, detergents can displace functionally relevant annular lipids. Thus, it has long been observed that many integral membrane proteins can denature and lose function once removed from the bilayer by detergent solubilization [6-12].

Several types of model membrane systems have therefore been developed that more closely mimic the lamellar lipid bilayer of native membranes. Proteoliposomes, synthetic lipid vesicles containing membrane proteins, provide such an environment. However, they can be heterogeneously sized and may promote protein aggregation. Further, their large size (up to several microns in diameter) makes them unsuitable for many spectroscopy techniques due to scattering or low rotation rates, and their vesicular structure precludes direct experimental access to both sides of the bilayer [13]. To address these technical issues, discoidal nanoscale lipid bilayers have been developed for membrane protein reconstitution. These include bicelles and nanodiscs. In bicelles, the rim of the lipid bilayer disc is stabilized by surfactants or by short-chain lipids [14,15]; in nanodiscs, the lipid discoid is bound by an amphipathic scaffolding protein [6,13]. These model membranes have been successfully used for both spectroscopic and structural studies [13,14,16-18]. However, bicelles require very specific combinations of lipids that may not be commensurate with the native bilayer and may not provide the optimal lipid environment for the protein of interest. Nanodiscs have no such specific lipid requirement; however, the reconstitution of membrane proteins into nanodics stabilized by a scaffolding protein requires an initial detergent solubilization step. Thus, it may be difficult to quantitatively remove the potentially destabilizing detergents following their assembly or to deconvolute the contribution of the scaffolding protein from the reconstituted membrane protein.

More recently, amphipathic polymers (amphipols) have emerged as a detergent-free means of membrane protein solubilization [6,15,19-21]. Amphipols likely form torroids around the transmembrane domains of membrane proteins, with the inner and outer polymer surfaces comprising nonpolar and polar pendant groups, respectively. Amphipols were originally developed to maintain membrane protein solubility after treatment with a nondenaturing detergent. The amphipols PMAL-B-100 and A8-35, for example, have been used to maintain the solubility [22] and catalytic activity [23] of a range of proteins, even very large (1.7 MDa) multisubunit complexes from mitochondrial membranes [24].

**Figure 1:**
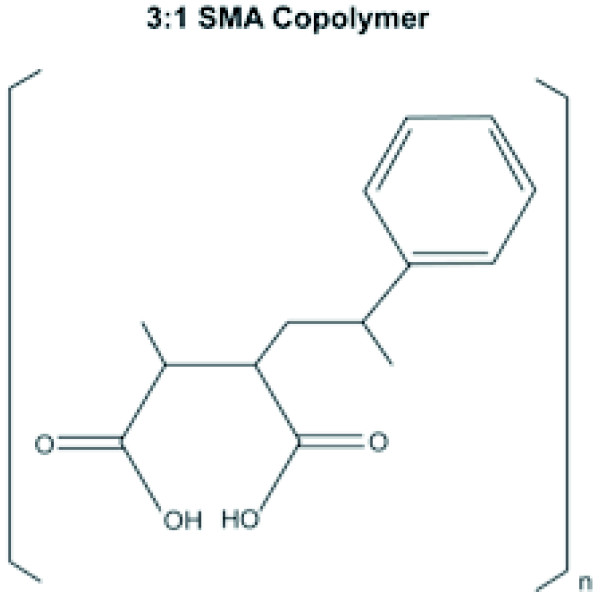
**The chemical structure of the SMA copolymer.** The 3:1 SMA copolymer consists of heterogeneously coupled hydrophobic styrene and hydrophilic maleic acid pendant groups.

Amphipols have not typically been used for the extraction of membrane proteins from lipid bilayers. However, a copolymer prepared from a 3:1 molar ratio of styrene to maleic acid (3:1 SMA, Figure 1) has been previously shown to solubilize lipids from vesicles of 1,2-dimyristoyl-*sn*-phosphocholine (DMPC), forming monodisperse disc-shaped polymer-lipid complexes 9–10 nm in diameter, termed Lipodisqs® [25]. This SMA copolymer has also been shown to extract the α-helical bundle protein bacteriorhodopsin [26,27] and the β-barrel protein PagP [27] from DMPC vesicles. The resulting lipid- and protein-containing particles had a larger diameter (11–12 nm) than empty discs and maintained the protein activity [26,27]. These investigations demonstrated that SMA could be used to extract membrane proteins that have been previously reconstituted into bilayers of synthetic phospholipids. They also highlighted the intriguing possibility that amphipols can be used in place of detergents for membrane protein reconstitution into soluble lipid nanoparticles. In the present study, we demonstrate for the first time that it is possible to extract functional membrane complexes directly from native biological membranes without prior detergent-mediated isolation or reconstitution. To this end, we tested whether the SMA copolymer could both disrupt membranes of mitochondria isolated from yeast (*Saccharomyces cerevisiae*) and extract functional membrane complexes.

## Results and discussion

### The SMA copolymer solubilizes native mitochondrial membranes

**Figure 2:**
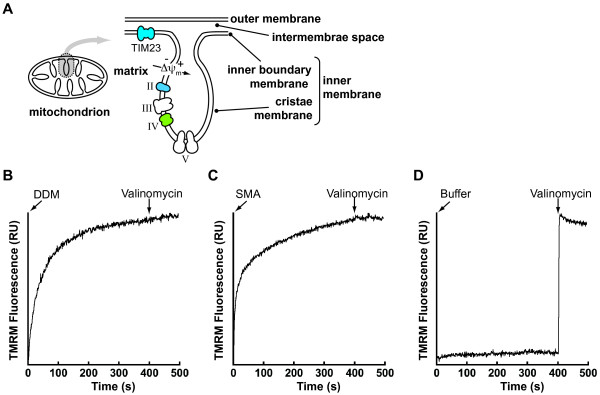
**Disruption of mitochondrial membranes by the SMA copolymer.** (**A**) Schematic of mitochondrial subcompartments and location of protein complexes analyzed in this study. Subunits of the TIM23 Complex and respiratory Complex II (cyan) were analyzed by immunodetection and respiratory Complex IV (green) was analyzed for enzyme activity. (**B-D**) Time course measurements of TMRM fluorescence were conducted with the addition of the following to actively respiring mitochondria: (**B**) DDM (final concentration 0.4% (w/v), (**C**) the SMA copolymer (final concentration 1 g SMA : 1 g mitochondrial protein), and (**D**) SMA buffer only. Valinomycin was added at the end of each time course to completely collapse the membrane potential.

Mitochondria contain two protein-rich membranes, with the topologically complex inner membrane (IM) subdivided into the inner boundary membrane and the cristae membrane (Figure 2A). The respiratory complexes of the IM generate a redox-coupled proton gradient across the IM, the majority of which is stored as a transmembrane electric potential (∆ψ_m_, 150–180 mV; Figure 2A) [28]. We first determined whether SMA could disrupt the IM by testing its ability to dissipate this ion gradient in actively respiring mitochondria. The relative ∆ψ_m_ can be assessed by the fluorescent potentiometric probe tetramethyl rhodamine methyl ester (TMRM), whose fluorescence intensity decreases with increasing membrane potential [29-31]. TMRM can thus be used as a reporter for membrane integrity, because an intact IM is required to generate and maintain a measurable ∆ψ_m_. As a positive control, we confirmed that the addition of detergent [n-dodecyl-β-D-maltoside (DDM)] dissipated the ion gradient across the IM as it solubilized the membranes (Figure 2B). Interestingly, the addition of the SMA copolymer dissipated the ion gradient to a similar extent as detergent, indicating that it also disrupted the integrity of the IM (Figure 2C). As a negative control, the addition of SMA buffer by itself had no effect on the mitochondrial membrane potential (Figure 2D). The K^+^ ionophore valinomycin was added at the end of each time course to cause the complete collapse of the ∆ψ_m_. Following DDM or SMA addition, there was no additional valinomycin-induced increase in TMRM fluorescence, indicating that the ion gradients had been completely collapsed in both cases. We therefore conclude that the SMA copolymer effectively disrupted the integrity of the IM.

### SMA extracts membrane proteins from the mitochondrial IM

**Figure 3:**
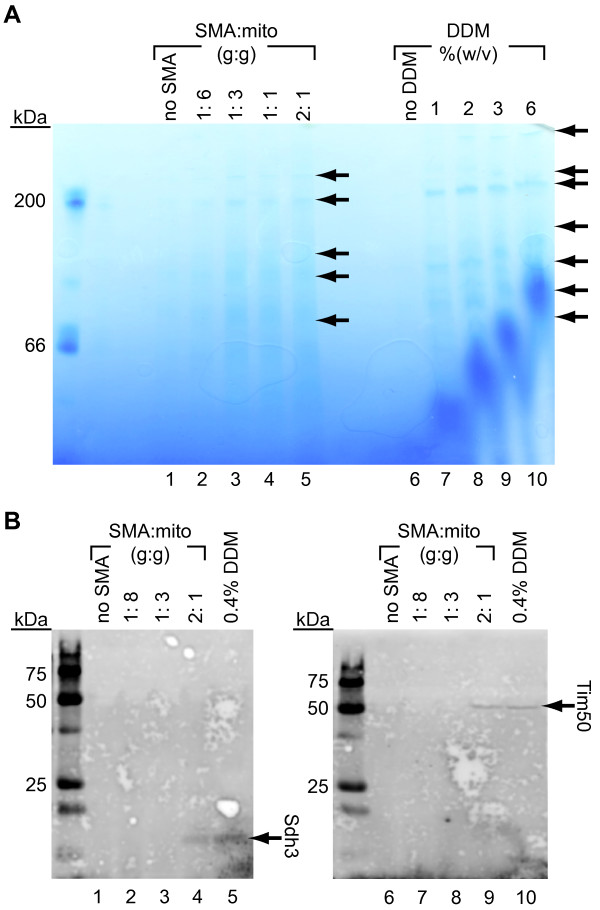
**Extraction of mitochondrial membrane proteins by the SMA copolymer.** (**A**) BN-PAGE analysis of proteins extracted from isolated mitochondria following incubation with increasing concentrations of SMA copolymer (lanes 1 to 5) or DDM (lanes 6 to 10). Arrows indicate locations of detectable bands. (**B**) Immunodetection of Sdh3 (left panel, lanes 1–5) and Tim50 (right panel, lanes 6–10) extracted from mitochondria by SMA and DDM at the concentrations indicated.

To analyze directly the ability of the SMA copolymer to extract mitochondrial membrane complexes, we first employed Blue Native Polyacrylamide Gel Electrophoresis (BN-PAGE) [32,33]. It has been amply demonstrated that multicomponent complexes solubilized from mitochondrial membranes with mild nonionic detergents such as DDM remain intact and can be resolved by native electrophoresis [34-37]. Thus, we subjected mitochondria to incubation with increasing amounts of DDM or the SMA copolymer, removed non-solubilized membrane by centrifugation, and analyzed the supernatants by BN-PAGE and Coomassie G-250 staining. We found that incubation of mitochondria with increasing amounts of SMA resulted in the extraction of large complexes (Figure 3A, lanes 1–5) that resolved as discrete bands with apparent molecular weights comparable to those solubilized by DDM (Figure 3A, lanes 6–10). Taken together, the results shown in Figures 2 and 3 confirm that the SMA copolymer can solubilize the native membranes of mitochondria, and does extract large protein complexes.

We next used Western blotting to demonstrate unequivocally that the SMA copolymer extracted integral membrane proteins from the mitochondrial membranes, and did not simply release proteins that were peripherally bound. Following incubation with SMA and DDM as above, mitochondrial extracts were resolved by denaturing gel electrophoresis and probed by immunoblotting with antisera against two integral membrane proteins: the Sdh3 subunit of respiratory Complex II (located in the cristae) and the Tim50 subunit of the TIM23 protein transport complex (located in the inner boundary membrane) (see Figure 2A). We found that both proteins were detected in mitochondrial extracts following incubation with DDM (Figure 3B, lanes 5 and 10 respectively) and with sufficient concentrations of the SMA copolymer (Figure 3B, lanes 4 and 9 respectively). Based on band quantification, the amount of Tim50 extracted by SMA at the concentration used was nearly equivalent (within 5%) to the amount extracted by DDM. By contrast, with the same SMA concentration, the amount of Sdh3 extracted was approximately 30% of that extracted by DDM. This difference in SMA extraction efficiency may be related to the location of the membrane protein within the morphologically complex inner membrane, and it suggests that SMA-based extraction conditions must be optimized on a protein-specific basis. Nonetheless, these results confirm that the SMA copolymer does extract integral membrane proteins from cellular membranes.

### SMA solubilization of mitochondrial membranes yields discoidal nanoparticles

**Figure 4:**
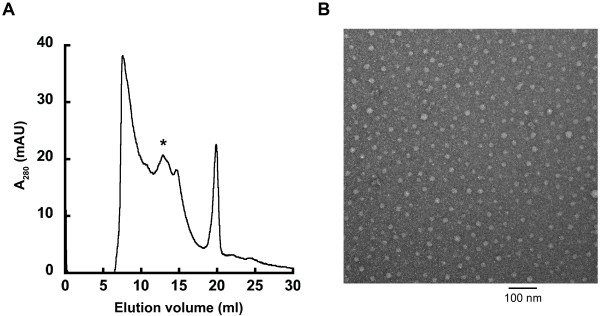
**The formation of discoidal SMA-bound nanoparticles.** (**A**) Size exclusion fractionation of SMA particles assembled with mitochondrial membranes. The chromatogram is absorbance at 280 nm to detect protein content. (**B**) Electron micrograph of mitochondrial-Lipodisqs® from the SEC fraction denoted by * (scale bar = 100 nm).

To assess the size and shape of SMA particles formed after incubation with mitochondria, we visualized the extracts by transmission electron microscopy (TEM) following fractionation by size exclusion chromatography (SEC). The gel filtration chromatogram of these extracts (Figure 4A) shows that particles formed from native mitochondrial membranes had a broad size range. This is in contrast to the monodisperse peaks previously observed following the extraction of single types of membrane proteins from synthetic bilayers [26,27]. However, our results are not unexpected given the large range of protein complex sizes that exist within the membranes of mitochondria and are consistent with the broad molecular weight range of extracted complexes detected by BN-PAGE (Figure 3A).

Samples from the fraction denoted by an asterisk on the SEC chromatogram were chosen for imaging because this fraction displayed robust mitochondrial enzymatic activity (see the following section). The TEM image of this fraction (Figure 4B) revealed a monodisperse population of SMA-stabilized discoidal nanoparticles consistent with those observed in previous reports [26,27]. We therefore conclude that the SMA copolymer can solubilize native cellular membranes and stabilize the bilayers as discoidal, polymer-bound particles. Hereafter these SMA-bound nanoparticles will be termed mitochondrial-Lipodisqs®.

### Respiratory Complex IV in SMA nanoparticles is functionally active

**Figure 5:**
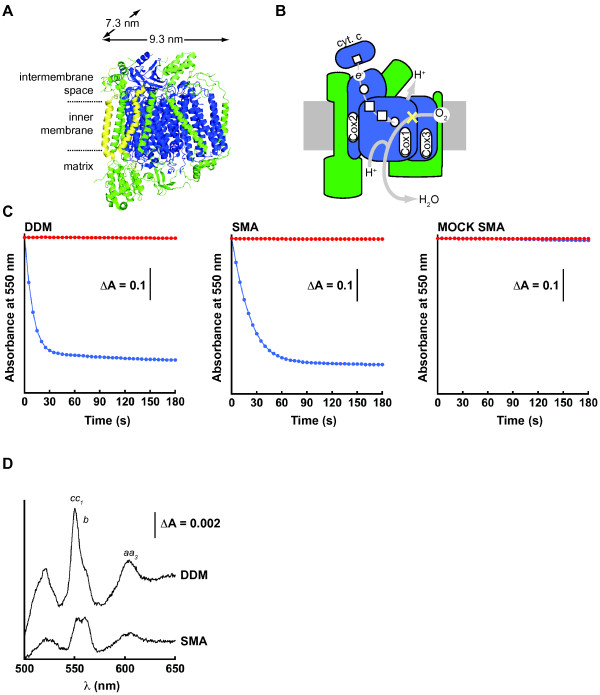
**Analysis of Complex IV enzymatic activity.** (**A**) Complex IV monomer structure from bovine (PDB 1 V54) with the catalytic core (blue), supernumerary subunits (green), and subunits absent in yeast (yellow). (**B**) Schematic of the Complex IV redox pathway with the color scheme as in panel A, showing redox centers [heme (square) and copper (circle)], paths of electron (thin grey line) and proton (thick grey line) transfer, and site of cyanide inhibition (yellow X). (**C**) Representative time courses of Complex IV catalytic activity monitored as the kinetics of ferrocytochrome c oxidation (decrease in absorbance at 550 nm) for DDM-solubilized mitochondria (left panel), SMA nanoparticles (center panel), and mitochondria subjected to mock SMA treatment (right panel). Reactions were performed in the absence of inhibitor (blue) or in the presence of potassium cyanide (red). (**D**) Representative difference spectra (reduced *versus* oxidized) of cytochromes in DDM-solubilized mitochondria (upper trace) and in SMA nanoparticles (lower trace). The positions of the α-absorption bands of cytochromes *cc*_*1*_, *b*, and *aa*_*3*_ are indicated. The 604 nm absorbance peaks of hemes *a* and *a*_*3*_, both coordinated by the Complex IV Cox1 subunit, were used to calculate the concentration of Complex IV in DDM- and SMA-treated samples.

To confirm the ability of mitochondrial-Lipodisqs® to support the function of extracted membrane complexes, we analyzed the activity of respiratory Complex IV (cytochrome c oxidase, Figure 2A) as a model. Complex IV consists of a catalytic core of three subunits (Cox1, Cox2 and Cox3) and multiple additional proteins (eight in yeast, ten in mammals) (Figure 5A) [38-41]. As the terminal enzyme of the electron transport chain, Complex IV catalyzes the oxidation of ferrocytochrome c and the reduction of molecular oxygen to water, pumping protons into the intermembrane space in the process (Figure 5B).

We assayed Complex IV activity spectrophotometrically by the decrease in absorbance at 550 nm resulting from the oxidation of pre-reduced cytochrome c. As shown previously (e.g., [37]) Complex IV maintained potassium cyanide (KCN)-sensitive activity following the solubilization of mitochondria by nonionic detergent [0.4% (w/v) DDM, Figure 5C, left panel]. We observed similar robust Complex IV activity in mitochondrial-Lipodisqs® formed upon incubation with the SMA copolymer (final concentration, 2 g SMA : 1 g mitochondrial protein, Figure 5C, center panel), confirming that the enzyme was active within those particles. To ensure that mitochondria-Lipodisq® preparations were free of contaminating membrane fragments that could potentially contribute to enzyme activity, all samples were carefully filtered (0.4 μm) following SMA solubilization (see Methods). To confirm further that our measured Complex IV activity originated from SMA-bound particles, we performed negative controls in which mitochondria were subjected to SMA buffer only, and no enzyme activity was found in those samples (Figure 5C, right panel).

To measure the specific cytochrome c oxidase activities in our DDM-solubilized and SMA nanoparticle samples, we measured the absorption bands of cytochromes *a* + *a*_3_ as an index of Complex IV concentration (Figure 5D). The α absorption peaks of cytochromes *aa*_*3*_ can be easily resolved from the α peaks of other respiratory chain cytochromes, thereby allowing a reliable measure of Complex IV in each sample [42-44]. Based on these measurements, we found that the specific Complex IV activity within mitochondrial-Lipodisq® preparations was comparable to that of DDM-solubilized mitochondria (Table 1).

**Table 1:** Specific cytochrome c oxidase activity of Complex IV

**Sample**	**Specific activity**
	**(nmol cyt. c min**^**-1 **^**pmol cyt. *aa3***^**-1**^**)**^**a,b**^
DDM (no KCN)	7.651 (2.232)
DDM (+ KCN)	0.002 (0.086)
SMA (no KCN)	6.358 (1.098)
SMA (+ KCN)	0.004 (0.033)

^a^Mean values are from a minimum of eight experiments from three independent preparations.

^b^Numbers in parenthesis are standard deviations.

As an additional means of confirming that Complex IV remained active within SMA-bound nanoparticles, we subjected mitochondrial-Lipodisqs® to SEC purification and measured the enzymatic activity of the fraction corresponding to that used for TEM imaging (Figure 4). This fraction displayed robust and cyanide-sensitive cytochrome c oxidase activity (Table 2), confirming the presence of active Complex IV within these particles.

**Table 2:** Cytochrome c oxidase activity of SEC-purified SMA particles

**Sample**	**Cytochrome c oxidation rate**
	**(μM cyt. c min**^**-1**^**)**^**a,b**^
no KCN	3.350 (0.503)
+KCN	0.017 (0.015)

^a^Mean values are from a minimum of three experiments from three independent preparations.

^b^Numbers in parenthesis are standard deviations.

An estimate of the dimensions of these mitochondrial-Lipodisqs® underscores the feasibility of reconstituting the multisubunit Complex IV holoenzyme into a single polymer-bound particle. The dimensions for the bovine homolog of Complex IV (the only high-resolution structure of the complex solved to date [38,39]) are approximately 9.3 nm × 7.3 nm (Figure 5A). The core subunits of the complex consist of a total of 14 helical transmembrane segments (Figure 5A, blue); in yeast, the supernumerary subunits contribute five additional transmembrane helices (Figure 5A, green). The dimensions of the mitochondrial-Lipodisqs® formed here (Figure 4B) are consistent with the previously reported diameter of ~12 nm (area ~113 nm^2^) [26]. Therefore, a single particle could easily accommodate one copy of Complex IV (area ~50 nm^2^). Further, assuming an average lipid cross-sectional area of 0.6 nm^2^, a discoid of this dimension could accommodate roughly 100 lipids per leaflet in addition to a single copy of the enzyme. These estimates of dimensions and lipid content are similar to those reported for the photoreceptor bacteriorhodopsin incorporated into Lipodisqs® [26].

Taken together, our results confirm that Complex IV maintains activity in SMA-bound particles. Therefore, while previous studies have demonstrated the activity of monomeric proteins (22 to 25 kDa) in Lipodisqs® [26,27], we show here, using Complex IV (ca. 220 kDa) as a model, that even large, multisubunit complexes can be reconstituted into these particles from cellular membranes in a fully operational state.

### Mitochondrial Lipodisqs® contain lipids from mitochondria

**Figure 6:**
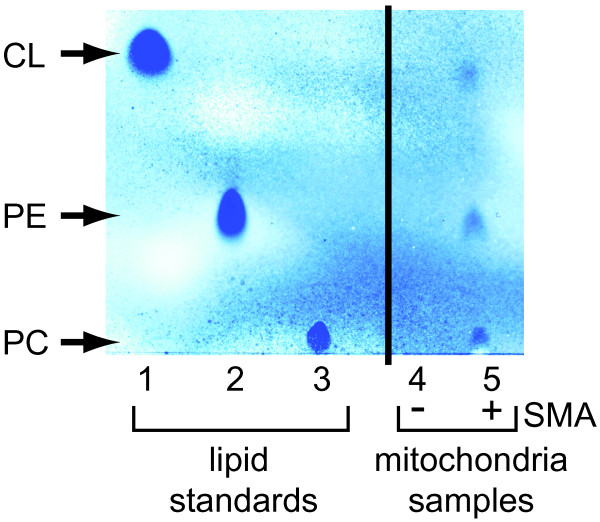
**Analysis of mitochondrial lipids contained in SMA particles.** Mitochondria subjected to mock SMA incubation (lane 4) or incubated with SMA to form mitochondrial-Lipodisqs® (lane 5) were analyzed for lipid content by thin layer chromatography. Lipid standards are shown in lanes 1–3.

It has been previously shown that monomeric proteins reconstituted from synthetic bilayers into Lipodisqs® are surrounded by lipids [26]. By thin-layer chromatography we confirmed the presence of the major mitochondrial lipids [phosphatidylcholine (PC), phosphatidylethanolamine (PE) and cardiolipin (CL)] in the mitochondrial-Lipodisqs® prepared in the present study (Figure 6, lane 5). By contrast, a control sample (SMA buffer only) revealed no residual lipids following our preparative steps (Figure 6, lane 4). These results indicate that lipids as well as intact complexes were extracted from the mitochondrial membranes and bound by the SMA copolymer and were thus present in the mitochondrial-Lipodisqs®. These results support the probability that native lipids are co-reconstituted into the SMA nanoparticles with the protein complexes.

## Conclusions

This study documents for the first time that Lipodisqs® containing isolated protein complexes from cellular membranes can be formed and used for biochemical and biophysical analyses. Specifically, we have shown that the SMA copolymer can disrupt native mitochondrial membranes and extract membrane proteins (Figures 2 and 3) and native lipids (Figure 6); that mitochondrial Lipodisqs® are SMA-bound discoids with dimensions similar to previously reported SMA nanoparticles [26,27] (Figure 4); and that the SMA copolymer can be used to isolate large multisubunit complexes in a fully-functional state (Figure 5, Tables 1 and 2). The mechanism by which the SMA copolymer fragments lipid bilayers into nanometer-scale discoids is not known. However, the process is highly pH-dependent, which may be related to conformational transitions of the hypercoiling SMA polymer that are dependent on the ionization states of its carboxylate groups [15,19].

By eliminating the requirement of a detergent-mediated step, the use of the SMA copolymer represents a significant advance in the study of native membrane complexes. As a tool for basic research, Lipodisqs® are an excellent means of analyzing membrane protein activities and interactions that may be lipid-dependent and sensitive to detergents. Additionally, these systems have potential in biophysical studies, in particular where single (or small numbers of) proteins are required. As a tool for practical applications, they have potential for the rapid characterization and diagnosis of dysfunctional membrane proteins taken directly from tissue samples of patients with suspected heritable diseases manifest in membrane proteins.

## Methods

### Preparation and solubilization of mitochondria

Mitochondria were isolated from *Saccharomyces cerevisiae* wild type strain D273-10B as described [45,46], resuspended in SIB buffer [20 mM HEPES-KOH (pH 7.5), 80 mM KCl, 5 mM MgCl_2_, 2 mM potassium phosphate (pH 7.5), 250 mM sucrose, and 0.3% (w/v) bovine serum albumin (BSA, fatty acid-free)], frozen in liquid nitrogen and stored at −80°C until use. For solubilization, mitochondria pellets were resuspended to a concentration of 1 mg mitochondrial protein ml^-1^ in either SIB buffer adjusted to pH 8.0 or SB buffer [50 mM Tris (pH 8.0), 200 mM NaCl]. For detergent solubilization, DDM was added to final concentrations ranging from 0.4 to 6.0% (w/v) as indicated. For SMA solubilization, lyophilized 3:1 SMA copolymer (Malvern Cosmeceutics Ltd, UK) was reconstituted as a stock of 25 mg ml^-1^ in 50 mM Tris (pH 8.0) as described [25] and added to samples at final concentrations ranging from 1 g SMA : 8 g mitochondrial protein to 2 g SMA : 1 g mitochondrial protein as indicated. For both DDM and SMA solubilization, samples were incubated at 26°C for 20 min, centrifuged (20,000 × g, 10 min, 4°C) and the resulting supernatants containing solubilized membrane proteins were collected. Further purification steps of SMA-treated samples for particular applications are described below.

### Measurement of membrane potential

The relative membrane potential (∆ψ_m_) of isolated mitochondria was assayed with the fluorescent potentiometric probe tetramethyl rhodamine methyl ester (TMRM, Molecular Probes) as described [47] using a Spex Fluorolog 3–22 spectroflorometer (Horiba Jobin-Yvon). Mitochondria were diluted to a concentration of 0.1 mg ml^-1^ in SIB lacking BSA with 100 nM TMRM (quenching mode) and kinetics measurements were taken (λ_ex_ = 547 nm, λ_em_ = 570 nm) with constant stirring of the sample. Prior to time course measurements, respiratory substrate (2.5 mM malate and 2.5 mM pyruvate) and 2 mM ATP were added to establish a maximal ∆ψ_m_. Subsequent additions included DDM at a final concentration of 0.4% (w/v), the SMA copolymer at a final concentration of 1 g SMA : 1 g mitochondrial protein, or an equivalent volume of SMA buffer only. At the end of each time course, the potassium ionophore valinomycin was added to a final concentration of 2.5 μM to completely collapse the ∆ψ_m_.

### Native gel electrophoresis and western blotting

For BN-PAGE analysis, mitochondria were prepared as above, but resuspended in 50 mM Tris (pH 8.0) prior to DDM and SMA solubilization. 5X BN-PAGE sample buffer [50 mM Trizma, 500 mM 6-aminohexanoic acid, 10% (v/v) glycerol, 2% (w/v) Serva Blue G] was added to supernatants, and 12 μl of each sample were loaded onto 4-15% precast polyacrylamide Mini-Protean TGX gels (BioRad). The cathode buffer contained 190 mM glycine, 23 mM Tris (pH 8.0) and 0.002% (w/v) Serva Blue G and the anode buffer consisted of 25 mM Trizma (pH 8.0) [33]. Following electrophoresis, gels were destained in 50% (v/v) methanol, 10% (v/v) glacial acetic acid and subsequently washed several times in water before visualization.

For immunodetection of mitochondrial proteins, samples were resuspended in SIB and solubilized with DDM or SMA as indicated. Supernatants were treated with equivalent volumes of 2X sample buffer [140 mM Trizma, 20% (v/v) glycerol, 4% (w/v) sodium dodecyl sulfate, 0.05% (w/v) bromophenol blue, 0.25 M dithiothreitol] and resolved by electrophoresis on 12% SDS-PAGE gels. Following transfer onto nitrocellulose membranes, Western blotting was performed with primary antibodies against the Sdh3 subunit of respiratory Complex II (Pacific Immunology) or against the Tim50 subunit of the TIM23 Complex (a gift from Dr. Dejana Mokranjac) and Amersham ECL Plex Cy5-conjugated secondary antibodies. Immunoblots were imaged on a BioRad Pharos Plus Molecular Imager using the Cy5 setting (635 nm laser excitation, 695 nm emission filter).

### Electron microscopy

For TEM imaging, SMA-solubilized mitochondria were filtered (0.22 μm) and purified by size exclusion chromatography on an AKTA Purifier system (GE Healthcare) using a Superdex 200 10/300 GL column equilibrated with 50 mM Tris (pH 8.0) and 200 mM NaCl. Purified samples were adsorbed for 30 sec onto carbon-coated, 400-mesh copper grids (Ted Pella, Inc.) made hydrophilic by glow discharge, washed with three drops of distilled water and stained with 1% uranyl acetate. Images were acquired using an AMT XR-40 (2048 × 2048 pixel) camera mounted on a Tecnai Biotwin G2 Spirit transmission electron microscope operated at 80 kV.

### Complex IV activity assays and spectrophotometric measurement of cytochromes

Respiratory Complex IV (cytochrome c oxidase) activity of solubilized samples was determined spectrophotometrically as described [48,49]. Equine cytochrome c (Sigma) was reduced in the presence of ascorbate, purified by gel filtration on a Sephadex G-50 column, and quantified by absorbance at 550 nm (ϵ_550nm_ = 27.6 mM^-1^ cm^-1^) [48]. To prepare samples for activity assays, mitochondria were incubated with 1% (w/v) DDM, with SMA at a concentration of 2 g SMA : 1 g mitochondrial protein, or with SMA buffer only (mock SMA incubation) and treated as described above. For SMA-solubilized and mock incubation experiments, samples were filtered through a 0.4 μm membrane to remove fragmented mitochondrial particulates that could contribute to enzyme activity. 120 μl reaction mixtures containing assay buffer [50 mM potassium phosphate (pH 7.4) and 50 μM EDTA] with 30 μM reduced cytochrome c were added to a quartz cuvette (path length 1 cm) and the reaction was initiated by the addition of 20 μl of mitochondrial sample. Cytochrome c oxidation kinetics were monitored by the reduction of absorbance at 550 nm on an Ultrospec 2100 pro spectrophotometer over 180 s with 5 s measurement intervals. Complex IV enzymatic rates were calculated from the difference in absorbance at 550 nm over the linear range (first 20 sec). For activity measurements of samples fractionated by gel filtration, SMA-solubilized particles were prepared as above and subjected to size exclusion chromatography on a Superdex 200 10/300 GL column equilibrated with assay buffer. Complex IV activity was measured as above except that 1 ml fractions were measured and 30 μM of reduced cytochrome c was added to initiate the reaction. In all cases, parallel reactions containing 4 mM potassium cyanide were assayed to confirm specific Complex IV activity.

To assay the cytochrome content of DDM- and SMA-treated mitochondria, samples were diluted 2X in buffer [50 mM Tris (pH 8.0) 200 mM NaCl] in a 1 ml quartz cuvette (path length 1 cm) and absorbance spectra were taken from 500 to 650 nm. Spectra of oxidized cytochromes of respiratory chain enzymes were recorded first, followed by spectra of fully reduced cytochromes in the presence of 3 mM sodium dithionite. The difference spectrum (reduced – oxidized) was used to calculate the concentration of cytochrome *a* + *a*_3_ of Complex IV by the peak of the alpha absorption band (604 nm) normalized by the isobestic point (630 nm) using the differential extinction coefficient value ∆ϵ_603-630nm_ = 24 mM^-1^ cm^-1^[42,43].

### Thin layer chromatography

Isolated mitochondria were incubated with SMA at a concentration of 2 g SMA : 1 g mitochondrial protein as above and subjected to filtration followed by incubation with a chloroform, methanol and water mixture (final volume ratio of 1:2:1.6) to extract lipids. Extraction was carried out for 1 h, followed by treatment with additional chloroform and salt (final volume ratio of 1:1:1.25). Phases were separated by low speed centrifugation and extracted lipids were then collected, dried down, and resuspended in 15 μl of chloroform. The entire volume was then spotted onto a silica gel plate (Analtech). As references, 1 μl each of POPC, POPE, and tetraoleoyl-CL (Avanti Polar Lipids) were also spotted. Plates were placed in a pre-equilbrated tank containing solvent (chloroform, ethanol, water and triethylamine at volume ratios of 30:35:4:32) for 1.5 h [50] and visualized by aerospray with molybdenum blue spray reagent (Sigma) [51].

## Abbreviations

SMA: Styrene maleic acid; amphipols: Amphipathic polymers; DMPC: 1,2-Dimyristoyl-*sn*-phosphocholine; POPC: 1-Palmitoyl-2-oleoyl-*sn*-glycero-3-phosphocholine; POPE: 1-Palmitoyl-2-oleoyl-*sn*-glycero-3-phosphoethanolamine; tetraoleoyl CL: 1’,3’-bis[1,2-dioleoyl-*sn*-glycero-3-phospho]-*sn*-glycerol; IM: Inner membrane; ∆ψm: Transmembrane electric potential; TMRM: Tetramethyl rhodamine methyl ester; DDM: N-dodecyl-β-D-maltoside; BN-PAGE: Blue native polyacrylamide gel electrophoresis; TEM: Transmission electron microscopy; SEC: Size exclusion chromatography; KCN: Potassium cyanide.

## Competing interests

The authors declare they have no competing interests.

## Authors’ contributions

AL participated in the conception and design of the study, conducted all laboratory experimentation except for TMRM assays and drafted the manuscript. CO assisted in BN-PAGE and Complex IV activity assays. KM performed the TMRM assays. CS participated in the design and implementation of the TLC experiments. AA and AW contributed to the conception and design of the study. NA coordinated the conception and design of the study, performed SEC and Complex IV activity assays and drafted the manuscript. All authors read and approved the final manuscript.
